# Diagnosis of premature rupture of membranes by assessment of urea and creatinine in vaginal washing fluid

**Published:** 2013-02

**Authors:** Nourossadat Kariman, Maryam Afrakhte, Mehdi Hedayati, Masoumeh Fallahian, Hamid Alavi Majd

**Affiliations:** 1 *Nursing and Midwifery Faculty, Ph.D. Candidate of Reproductive Health, Shahid Beheshti University of Medical Sciences, Tehran, Iran.*; 2 *Faculty of Shahid Beheshti Medical School, Shohadaye Tajrish Hospital, Tehran, Iran.*; 3 *Obesity Research Center, Research Institute for Endocrine Sciences, Shahid Beheshti University of Medical Sciences, Tehran, Iran.*; 4 *Faculty of Shahid Beheshti Medical School, Ayatollah Taleghani Hospital, Tehran, Iran.*; 5 *Faculty of Shahid Beheshti, Para Medical School, Tehran, Iran. *

**Keywords:** *Premature rupture of membranes*, *Creatinine*, *Urea*, *Vaginal washing fluid*

## Abstract

**Background: **Rupture of fetal membranes can occur at any gestational age. Premature rupture of membranes (PROM) means rupture of fetal membranes before the onset of labor.

**Objective:** The purpose of this study was to evaluate and compare the reliability of the vaginal washing fluid urea and creatinine for the diagnosis of PROM and to determine cut-off values.

**Materials and Methods:** A total of 179 pregnant women were recruited. All patients underwent different examinations. These included nitrazine paper test, fern test, amniotic fluid pooling, vaginal washing fluid urea and creatinine sampling. The one group consisted of 126 pregnant women between 14 and 41 weeks of gestation with the complaint of vaginal fluid leakage. Patients who had positive pooling, nitrazine paper test and fern test were considered as confirmed PROM group (group 1). On the other side, patients with pooling (-) and/or nitrazine paper test (-) and/or fern test (-) were taken as suspected unconfirmed PROM cases (group 2). The control group consisted of 53 pregnant women between 14 and 41 weeks of gestation without any complaint or complication. Weconducted one-way ANOVA test on the urea and creatinine measures and post-hoc comparison test. Cut-off value was determined by receiver operating characteristic (ROC) curve.

**Results:** Vaginal fluid concentrations of urea and creatinine were significantly different between the three groups (p<0.001). The sensitivity, specificity, positive and negative predictive values and accuracy were all 100% in detecting premature rupture of membranes by evaluation of vaginal fluid creatinine concentration with a cut-off value of 0.45 mg/dl, respectively.

**Conclusion:** This study demonstrates that of two markers investigated creatinine has the higher diagnostic power.

## Introduction

Premature rupture of membranes (PROM) refers to rupture of the fetal membranes prior to the onset of labor and can occur at any gestational age even at 42^nd^ week (1-4). PROM has previously been reported to occur in 8-19.53% of term pregnancies and 2-25% of all pregnancies (3-7). 

Besides, Nili and Shams Ansari reported a PROM prevalence of 7% in Vali-e-Asr Hospital of Tehran, Iran (8). PROM has been shown to be the cause of 18-20% of prenatal mortalities and 21.4% of prenatal morbidity (5, 9-11). Compared with normal group, the average hospitalization period of term and preterm newborns with PROM were prolonged 20% and 25.1% respectively. Consequently, the average costs of hospitalization were increased 30.5% and 60% respectively (11). Maternal complications include clinically evident intra-amniotic infection which occurs in 13-60% of women with PROM in comparison with 1% prevalence of term and postpartum endometritis (6, 12). 

“PROM is a clinical diagnosis actually. It is typically suggested by a history of watery vaginal discharge and is confirmed on sterile speculum examination” (3). The traditional minimally invasive gold standard for diagnosis of PROM relies on clinician’s ability to document three clinical signs on sterile speculum examination: 1) visual pooling of clear fluid in the posterior fornix of the vagina or leakage of the fluid from the cervical os; 2) an alkaline pH of the cervico-vaginal discharge, which is typically demonstrated by nitrazine paper (whether the discharge changes nitrazine paper from yellow to blue); and/or 3) microscopic ferning of the cervico-vaginal discharge (3, 4, 12). Diagnosis of PROM is easy in the presence of obvious rupture of membranes while several numbers of false positive and negative results obtained through applying conventional diagnostic methods in the suspected cases of PROM may result in inappropriate interventions such as hospitalization and induction of labor (13-16). 

History has been shown to be reliable only in 10 to 50 percent of patients in order to diagnose PROM (13, 14). Although inspection of fluid leakage from cervix has been traditionally the only method for definite diagnosis of PROM, it is associated with 12-30% false negative results (17). Nitrazine test may also lead to false positive or negative results due to probable contamination by alkaline urine, semen, blood, meconium, vaginitis, cervicitis and using antibiotics. Fern test has also 13-30% false negative and 5-30% false positive results (13, 14).

“Several studies have been conducted to find a definite, easy, noninvasive and reliable diagnostic test for PROM in recent years” (18). These studies have mainly focused on biochemical agents with high concentration in amniotic fluid. Prolactin, alpha-fetoprotein (AFP), insulin like growth factor binding protein (IGFBP-1), fetal fibronectin (fFN), Lactat and beta-subunit of human gonadotropin (β-HCG) have been mentioned as some of these factors (7, 13, 14, 19-29). 

However, results of using aforementioned tests have been variable (29, 30). Recently, the focus has been on urea and creatinine in cervicovaginal discharge. These studies reported the accuracy of urea and creatinine to determine the PROM from 90-100% (29-31).

“Urea plays an important role in the metabolism of nitrogen-containing compounds in the urine” (32). Creatinine is a break-down product of creatinine phosphate in muscles and is usually produced at a fairly constant rate and is mainly filtered out of the blood by kidneys (33). Urea and creatinine of fetal urine are the most important sources of amniotic fluid in second half of pregnancy (31). Thus we hypothesized that vaginal fluid creatinine and urea may be helpful in diagnosis of PROM. 

Indeed, the aim of this study was to evaluate and compare the reliability of vaginal washing fluid urea and creatinine for diagnosis of PROM and to determine cut-off values.

## Materials and methods

In this Laboratory diagnostic analytical study, sampling was performed by non-probability (convenience) method and sample size was determined based on the prevalence of PROM (5%), =0.05 and ε=0.2. This diagnostic study has been performed to evaluate a diagnostic test for PROM between May 2008 and September 2009 in prenatal clinic and delivery ward of Taleghani Hospital, Tehran, Iran. 

Women with singleton pregnancy and gestational age between 20-42 weeks were studied. Subjects with meconium in amniotic fluid, visible blood in vaginal secretion, intercourse in the prior night, use of vaginal drugs, presence of fetal anomalies, regular uterine contractions, intrauterine fetal death and prenatal complication were excluded. Among 185 pregnant women who were admitted with the complaint of vaginal fluid leakage between 14 and 41 weeks of gestation, 126 cases were included through non-probability (convenience) sampling in the present study. 

The sample size was determined based on =0.05, =0.2 and the prevalence of PROM (0.05). The remaining 59 pregnant women were excluded due to the presence of fetal anomalies, intrauterine fetal death, known disease, prenatal complication, and visible blood in vaginal secretion, use of vaginal drugs or intercourse in the prior night, meconium in amniotic fluid, multiple pregnancies and regular uterine contractions. 

Demographic and obstetric characteristics, results of speculum examination, fern test, nitrazine test, urea and creatinine were documented by the researcher according to a data form, validity of which was confirmed by content validity method. Urea concentration was measured by enzymatic photometry or urease, and creatinine concentration was determined by Jaffee synthetic chemical calorimetric. Calibration was used to confirm validity of urea and creatinine measuring methods. The reliabilities of data form and speculum physical exam were confirmed by test-retest and reliability of enzymatic photometry or urease, Jaffee synthetic chemical colorimetric, fern and nitrazine tests were established by inter-rater consistency.

This study was approved by ethics committee of Shahid Beheshti University of Medical Sciences and written informed consent was obtained from all participants. Gestational age was determined based on the first day of last menstruation period in reliable cases, or one ultrasound in less than 14 weeks or two ultrasound documents between 14 and 24 weeks of pregnancy. Pregnant women were examined in lithotomy position, leakage of fluid was inspected by sterile speculum and results were registered as positive, negative or suspicious. A cotton tip applicator was inserted in deep vagina and was immediately transferred on nitrazine paper. 

PH above 6.5 was considered positive. A sample of cervicovaginal secretion was taken by a similar method and was expanded on slides. The slides were examined after drying by microscope (10 magnification) for diagnosis of ferning pattern. Patients who had positive pooling, nitrazine paper test and fern test were considered as confirmed PROM group (group 1). On the other side, patients with pooling (-) and/or nitrazine paper test (-) and/or fern test (-) were taken as suspected unconfirmed PROM cases (group 2). 

From 126 patients, 60 patients who fulfilled the criteria were included in group 1 and the remaining 66 patients were included in group 2. Meanwhile, among pregnant women admitted to prenatal clinic for their regular prenatal control visit, 53 pregnant women with 14-41 weeks of gestational age without any complaint or complication and with pooling (-), nitrazine paper test (-) and fern test (-) were taken as control group (group 3). Procedures described before were applied to control group as well. 

Thereafter, vaginal washing fluid urea and creatinine sampling was performed as follows: 5 ml of sterile normal saline was injected into the posterior fornix of vagina and then was aspirated by the same syringe and was sent immediately to the laboratory. All speculum examinations were performed by the same obstetrician and all samples were studied in Research Institute for Endocrine Sciences Laboratory (which is located in Taleghani Hospital), by the same technique and the same technician in order to eliminate inter-observer sampling difference. 

Furthermore, diagnosis of PROM was confirmed by AFI (Amniotic Fluid Index) through ultrasound examination by the resident of radiology. Cut-off value was determined by receiver operating characteristic (ROC) curve. All the speculum examinations were done by the same obstetrician and all the samples were studied by a laboratory expert and by the same technique in order to eliminate inter-observer sampling difference. 

**Statistical analysis**

Statistical analysis was performed by statistical package for the social sciences (v.16) software. Results have been expressed as frequency, mean and standard deviation. Chi^2^ and one-way ANOVA test were used to compare groups with each other. P>0.05 was considered statistically significant.

## Results

Demographic data for each group are presented in table I. The parameters (age, gestational age, gravid and parity) were compared with analysis of variance between groups test. There were no statistically significant differences between groups (p>0.05). Most of the patients were housewives (groups1=91.66%, group 2=87.87%, group 3=92.45%). No statistically significant difference has been observed between these groups with respect to these factors (p=0.9).

Table II shows the mean concentrations of vaginal fluid urea and creatinine among groups. The mean vaginal fluid urea levels in group 1, 2 and 3 were 13.77±5.41mg/dl (range 1.0-43), 4.71±3.64 mg/dl (range 0.2-32) and 5.13±5.97 mg/dl (range 0.1-23) respectively, and the differences were statistically significant (p<0.001). Furthermore, the mean vaginal fluid creatinine levels in group 1, 2 and 3 were 1.58±1.01 mg/dl (range 0.5-7.2), 0.36±0.23 mg/dl (range 0.1-1.1) and 0.22±0.10 mg/dl (range 0.1-0.4) respectively. The differences between groups were statistically significant (p<0.001).

**Figure 1:**
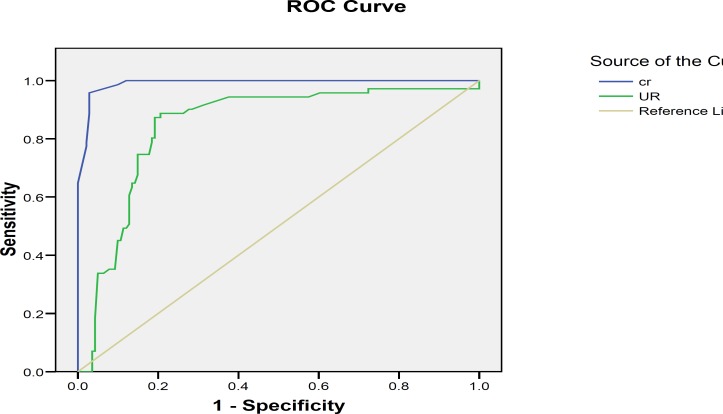
Receiving operator characteristic curve for vaginal urea and creatinine Levels

Receiver operating characteristic (ROC) curve analysis was used to establish the optimal cut-off concentrations for vaginal washing fluid urea and creatinine. From the ROC curves, 0.45 mg/dl was set as a cut-off value for creatinine and it is found that a cut-off value of 6.0 mg/dl is optimal for urea (Figure 1). 

The areas under the curves are 99.99% for creatinine and 84% for urea. According to the urea cut-off point sensitivity of 90%, specificity of 79%, positive predictive value of 83%, negative predictive value of 87.5% and accuracy of 85% were found. Besides, the sensitivity, specificity, positive predictive value, negative predictive value and accuracy were all 100% in detecting PROM by evaluating vaginal fluid creatinine concentration with a cut-off value of 0.45 mg/dl.

**Table I:** The demographic characteristics of groups

	**Group 1** **(PROM) (n=60)**	**Group2** **(Suspected) (n=66)**	**Group3** **(Control) (n=53)**	**p-value**
Age (year)	26.25 5.40	25.46 6.0	25.54 4.69	0.85
Gestational age (week)	38.23 2.42	38.40 2.79	38.05 3.69	0.37
Gravida	1.76 1.29	1.83 1.2	1.75 1.06	0.10
Parity	0.58 1.76	0.57 0.82	0.6 0.9	0.40

The difference between groups tested with one way ANOVA (age, gestational age, gravida and parity).

**Table II:** Vaginal fluid urea and creatinine level (ml/dl) among groups

	**Group 1** **(PROM) (n=60)**	**Group** **2** **(Suspected) (n=66)**	**Group 3** **(Control) (n=53)**	**p-value**
Urea (mg/dl)	13.77 5.41	4.71 3.64	5.13 5.97	0.001
Creatinine (mg/dl)	1.58 1.01	0.36 0.23	0.22 0.10	0.013

The difference between groups tested with one way ANOVA and compared with Scheffe multiple comparison tests

## Discussion

As mentioned before, a timely and accurate diagnosis of PROM is critical to optimize perinatal outcome and minimize serious complications such as cord prolapse and infections including chorioamnionitis and neonatal sepsis (2, 34, 35). In most cases diagnosis is made according to the clinical complaints and traditional methods (13). However, clinical complaint of patient is not reliable (14). 

In this regard, in the present study only 60 patients from 126 pregnant women with fluid leakage complaint had confirmed PROM, while PROM could not be confirmed in 66 of them with traditional diagnostic techniques. With the possible exception of direct visualization of amniotic fluid spurting from the cervical os, all clinical signs have limitations in terms of diagnostic accuracy, cost and technical case. 

Moreover, reliance on clinical assessment alone leads to both false-positive and false-negative results (3). Thus, we need simple, reliable and rapid tests for diagnosis of PROM. Since there is no unique and noninvasive gold standard test applicable to all patients with 100% accuracy several biochemical markers have been studied previously (14). Despite the improved diagnostic value of these markers, they have not become popular because of their complexity and cost (13).

As far as we know, Five studies related to PROM and vaginal washing fluid urea and Urea-creatinine have been published so far. The first study was conducted by Li Hy *et al* (29). “The purpose of this study was to determine the usefulness of vaginal fluid hCG, AFP and creatinine measurements in detection of PROM. In that study 10 control patients and 10 confirmed PROM cases were included and the results showed that the sensitivity, specificity, positive predictive value, negative predictive value and accuracy of creatinine were 90%, 100%, 100%, 90.9%, and 95% respectively”(29). 

They found that creatinine in vaginal fluid washings is a useful marker for PROM diagnosis. It was less expensive and easier to measure than hCG and AFP, and appeared to be more accurate than hCG. The second study was reported by Gurbuz *et al* (36). The study group consisted of 54 women in their third trimester of pregnancy with the diagnosis of PROM established by inspection of vaginal pooling while the control group consisted of 34 pregnant women with intact membranes. 

A cut-off value of 0.12 mg/dl was proposed for Creatinine and its sensitivity, specificity, positive predictive value, negative predictive value and accuracy in detecting PROM based on the aforementioned cut-off value were calculated to be 100%. They concluded that vaginal creatinine measurement is cheaper and faster than other methods, and has higher sensitivity and specificity to establish accurate diagnosis.

The third study, which was the first to use urea for PROM diagnosis was carried out by Kafali and Oksuzler (31). In that study 47 patients with confirmed PROM, 36 patients with suspected but unconfirmed PROM and 56 pregnant women without any complaint or complication were included. “The sensitivity, specificity, positive predictivity and negative predictivity were all 100% in detecting PROM by evaluation of vaginal fluid urea and creatinine concentration with cut-off values of 12 and 0.6 mg/dl respectively”(29). 

The fourth study related to vaginal washing fluid urea and creatinine levels was conducted by Kariman *et al* (37). 84 pregnant women in two groups, 42 confirmed PROM and 42 controls were included. The mean level of vaginal fluid urea and creatinine in the PROM group was significantly higher than the intact fetal membranes group. They speculated that in the absence of urine and macroscopic bloody contamination, measurement of urea and creatinine of cervicovaginal washing- fluid confirms an accurate diagnosis of PROM.

The other study was carried out by Mohamed and Mostafa (2011). The sensitivity, specificity, positive predictivity and negative predictivity were 100% in detecting PROM by evaluation of vaginal urea and creatinine concentration with cut-off values of 13.2 mg/dl and 0.31 mg/dl respectively (38). In the present study, we determined a cut-off value of 0.45 mg/dl for creatinine. We have found that power diagnostic including sensitivity, specificity, positive predictive value, negative predictive value and accuracy of vaginal fluid creatinine was 100%. The optimal cut-off value for urea (6 mg/dl) gave a sensitivity level of 90%, at a specificity of 70.0%, positive predictive values of 83.0%, and negative predictive values of 87.5% and accuracy level of 85%. 

The analysis of data revealed that creatinine has a higher diagnostic power for predicting PROM than urea. In this study, three tests including direct speculum examination, fern test and nitrazine test were applied for diagnosis of PROM. Moreover, inclusion criteria were so that interfering factors of these tests could be controlled. Another strength point of this study is investigating patients in three groups (confirmed PROM, suspected but unconfirmed and control group).

Urea is present in amniotic fluid, maternal blood and urine. “In the first half of pregnancy creatinine concentrations are similar in maternal serum and in amniotic fluid” (39). “Pregnant women in the first gestational group have a mean creatinine concentration of 0.6 mg/dl in the amniotic fluid, similar to which is found in maternal serum” (31, 39). Oliveira *et al* have observed significant correlations between gestational age and amniotic fluid creatinine (r>0.85, p<0.01) (40). 

Meanwhile, “Creatinine concentrations in amniotic fluid increased gradually between 20-32 weeks of gestation and more rapidly thereafter, when they were two to four times higher than in maternal serum” (31, 40). Our study reported low vaginal creatinine and urea in control group pregnant women with intact amniotic membranes. After rupture of fetal membranes a high level of creatinine can be detected in vaginal fluid discharge.

## Conclusion

In conclusion, our study demonstrated that the measurement of vaginal fluid urea and creatinine is a simple and reliable test for diagnosis of PROM. Furthermore, creatinine assay with higher sensitivity and specificity is a possible candidate to become the gold standard diagnostic test for PROM.
